# Mixed martial arts athletes demonstrate different brain vital sign profiles compared to matched controls at baseline

**DOI:** 10.3389/fneur.2024.1438368

**Published:** 2024-09-12

**Authors:** Thayne A. Munce, Shaun D. Fickling, Shaquile R. Nijjer, Daniel N. Poel, Ryan C. N. D’Arcy

**Affiliations:** ^1^Environmental Influences on Health and Disease Group, Sanford Research, Sioux Falls, SD, United States; ^2^Department of Pediatrics, University of South Dakota Sanford School of Medicine, Sioux Falls, SD, United States; ^3^Centre for Neurology Studies, HealthTech Connex, Surrey, BC, Canada; ^4^BrainNET, Health and Technology District, Surrey, BC, Canada; ^5^DM Centre for Brain Health, Faculty of Medicine (Radiology), University of British Columbia, Vancouver, BC, Canada

**Keywords:** event-related potential (ERP), brain vital signs, MMA, subconcussion, radar plot

## Abstract

We investigated objective brain vital signs derived from event-related potentials (ERPs) for mixed martial arts (MMA) athletes and matched controls (*N* = 24). Brain vital sign scans were acquired from 9 MMA athletes and 15 age-and sex-matched controls. Our analysis specifically compared differences in brain vital signs between MMA athletes and controls at baseline. We predicted that MMA athletes would show significant differences relative to controls due to their ongoing exposure to repetitive head impacts. Participants were scanned to extract three well-established ERPs: N100 for auditory sensation; P300 for basic attention; and N400 for cognitive processing. Scans were verified using automated reports, with N100, P300, and N400 amplitudes and latencies manually identified by a blinded reviewer. Brain vital signs were compared across groups with a Kruskal-Wallis H-test for independent samples, with FDR correction for multiple comparisons. We identified significant differences between MMA athletes and controls. Specifically, there were significant N400 amplitude reductions, indicating that exposure to repetitive head impacts in MMA may be associated with changes in brain function.

## 1 Introduction

Mixed martial arts (MMA) is a combat sport that continues to gain worldwide popularity. In competition, MMA athletes attempt to knock out, submit or out-point their opponent through a variety of striking, grappling and submission techniques. Through regular training and competition over many years, MMA athletes are routinely exposed to an undetermined amount of brain trauma, increasing their risk for concussion and other types of brain injury (1).

Recent evidence has demonstrated that contact-sport athletes are not only threatened by concussions, but also subconcussive brain trauma associated with the frequency of repetitive head impacts (2, 3). While subconcussion is often imperceptible, as it does not have any recognizable clinical signs or symptoms, neurological and/or neuromuscular function may be impaired, which may limit performance and leave the athlete more susceptible to subsequent injury (4). In the long term, exposure to repetitive, subconcussive head impacts has been linked to the onset of neurodegenerative disorders such as chronic traumatic encephalopathy (CTE) (5, 6). Electroencephalography (EEG)-derived event-related potentials (ERPs) (7), which represent brain responses to specific stimulus events, have increasingly been applied as objective, physiological measurements of cognitive function (8)_._ To translate this capability to the point-of-care, we developed and validated the brain vital signs framework (9). The brain vital signs approach extracts three well-established target ERP responses: the N100 as a measure of auditory sensation (10); the P300 as a measure of basic attention (10); and the N400 as a measure of cognitive processing (11). All three responses are elicited from a rapid auditory stimulation sequence comprised of randomly distributed auditory tones and spoken word pairs (9). Each response is evaluated in terms of latency (milliseconds) and amplitude (microvolts) relative to standardized normative data and mapped as six metrics on a radar plot, where a symmetric hexagon shape represents a cognitive profile within the range of healthy norms. Together, the N100, P300 and N400 measurements of brain function provide enhanced sensitivity through objective neurophysiological measures to track cognitive changes in the brain.

Brain vital sign monitoring has recently been utilized as a sensitive measure for subconcussive impacts in contact sports. In an initial study by Fickling et al. of acute concussion in Junior A ice hockey players, an exploratory examination detected significant pre-versus post-season delay in N400 latency that was suggestive of delayed cognitive processing speed due to exposure to contact over the course of the season (12). These findings were subsequently replicated in two groups of ice hockey players (Junior A and Bantam) as well as in youth tackle football players (12–14). These follow up studies demonstrated a significant linear relationship between brain vital signs changes and measures of head impact exposure. It was further shown that the changes in brain vital signs were significantly predictive of the total number of impacts that a player received, as measured by head-mounted accelerometers. In addition, brain vital signs changes were also significantly related to the total number of contact sport sessions (including games and practices) in which players participated. Collectively, these results, along with the emerging literature, indicate that exposure to repetitive subconcussive head impacts in a variety of contact sports is associated with measurable changes in brain function. However, it is unknown if similar subconcussive changes are concomitant with MMA participation.

## 2 Objectives

The study objectives were to investigate subconcussive changes in brain vital signs in MMA athletes compared to matched controls at baseline. Our hypothesis predicted that MMA athletes would show significant differences in brain vital signs relative to controls due to their greater exposure to repetitive head impacts.

## 3 Materials and methods

### 3.1 Participants

Overall, 34 participants were enrolled in the study, which was approved by Institutional review/ethics boards at Sanford Health and Advarra. There were 15 MMA athletes (*N* = 15, Age = 25.07 ± 2.41, 2 female, 13 male) and 19 control participants (*N* = 19, Age = 26.21 ± 3.11, 3 female, 16 male). Participants in the Control group were matched as closely as possible in relevant characteristics (e.g., age, sex, fitness level). Each participant provided written consent, according to the declaration of Helsinki. Inclusion criteria for participant recruitment was as follows: (1) MMA athletes: Adults (≥ 18 years of age) who were currently training (minimum of 3x/wk) in MMA and had been training for at least six months prior to study participation; (2) Control group: Age and sex matched adults (≥ 18 years of age) who were currently physically active (exercised for a minimum of 3x/wk. at a moderate to high intensity).

The experimental design was a longitudinal, repeated-measures cohort study. Where applicable, baseline assessments were done at least 90 days from a previous fight (MMA group only) and 90 days (MMA) or 1 year (control) following medical clearance from a previously diagnosed concussion. Three MMA fighters reported a previously diagnosed concussion, with the most recent one occurring about 4.5 years before baseline testing. Additionally, nearly half of the control participants reported a previous concussion, with the most recent one approximately 21 months before enrollment. No participant reported a concussion within 20 months of enrollment. Therefore, the acute effects of a recent concussion were not expected to influence the results of this study. While not the focus of the current analysis, the MMA group received up to three follow-up scans after a fight was completed.

Inconsistent fight schedules, participant compliance, and technical issues with data collection resulted in sample size attrition. Due to missing data and unequal samples, a within-subject, repeated-measures analysis was not possible. Accordingly, the primary analysis focused on between-group comparisons of MMA athletes and controls at baseline. There were 24 participants who successfully completed a baseline scan: 9 MMA athletes (*N* = 9, Age = 25.67 ± 2.17, 0 female, 9 males) and 15 controls (*N* = 15, Age = 26.07 ± 3.28, 3 female, 12 male). Two MMA athletes and four controls completed two baseline scans for purposes distinct from the current investigation. In cases of multiple baselines, analyses were completed using only the first successful scan.

### 3.2 Brain vital signs data collection

Brain vital signs were extracted from ERPs using a g. Nautilus EEG cap (Gtec Medical Engineering, Austria) with three embedded electrodes (locations were Fz, Cz, and Pz). Midline electrodes were chosen based on the central distribution of all three responses, which can shift along the anterior/posterior and left/right axes, but is most reliably measured along the midline. It has been shown that Fz, Cz, and Pz can robustly measure all three ERP components (15), and can enable faster set-up times. After putting the cap on the participants head, g.GAMMAsys electrode gel was injected at each location for conductivity. A reference electrode was clipped to the right earlobe and disposable Ag/AgCl electrodes were used for ground (forehead), and electro-oculagram (EOG) recording from the supra-orbital ridge and outer canthus of the left eye. Skin-electrode impedances were maintained at <30k impedance at each site. A predefined ~6 min auditory stimulus sequence was used, in which acoustic stimuli (interwoven tones and word pairs) were delivered binaurally through earphones. This was a passive task and subjects were instructed to pay attention to the auditory stimuli while maintaining visual fixation on a cross located 2.0m away in a closed, quiet room. The same facility was used for all scans.

### 3.3 Brain vital sign data processing

Raw EEG data were processed at the individual level using standard analysis methods. Data from three electrodes (Fz, Cz, & Pz) were sampled at 500Hz and bandpass filtered (0.1-20Hz). Ocular correction was applied using an adaptive filter (16) with the EOG recordings as a reference. Denoised, filtered data were then segmented into epochs around common stimulus types (Epoch length: -100ms pre-stimulus to +900ms post stimulus). Epochs were grand averaged across all electrodes to generate a single composite evoked potential waveform for each stimulus type (standard tone, deviant tone, congruent word, incongruent word). To identify each brain vital sign (peak amplitude and latency), we concentrated on the 50-150ms, 250-350ms, and 350-450ms windows to select the N100, P300, and N400, respectively. We also examined responses just outside these windows. Response size (peak amplitude in microvolts) and timing (peak latency in milliseconds) were manually verified and recorded by an experienced blinded reviewer (17). N100 and P300 were labelled on the deviant tone response, and the N400 was labelled only on the incongruent word response. Overall, we collected 264 standard and 24 deviant tone trials, 36 congruent and 36 incongruent word pair trials. Epochs that still contained noise after bandpass filtering and ocular correction were rejected. If less than 66% of the standard and deviant tone responses remained after rejection, we did not use that dataset in our analysis. The congruent and incongruent epochs were similarly thresholded at 75%.

### 3.4 Statistical comparisons

Statistical analyses consisted of a time-series analysis of the amplitudes and latencies for the N100, P300, and N400. Group comparisons used a Kruskal-Wallis H-test for independent samples, with Benjamini-Hochberg Method of False Discovery Rate correction for multiple comparisons. We compared MMA athletes versus controls at baseline (Table 1).

**Table 1:** Brain vital sign measures for MMA athletes and controls across comparisons.

		h	*p* (uncorrected)	*p* (FDR corrected)
Baseline comparison	N100 amplitude	1.4325	0.2314	0.3470
	N100 latency	1.5172	0.2180	0.3470
	P300 amplitude	2.1342	0.1440	0.3470
	P300 latency	0.1507	0.6978	0.6978
	N400 amplitude	7.0440	0.0080	0.0477*
	N400 latency	0.9680	0.3252	0.3902

Bold values indicate significance (*p < 0.05).

### 3.5 Radar plot comparison

Standardized radar plots were generated to visualize multivariate changes in brain vital signs (N100, P300, and N400 responses) For any given response, larger amplitudes and faster (i.e., smaller) latencies are plotted on the peripheral of the figure. Normative (i.e., group median) results converge on a symmetric hexagonal profile. The reference range (ref range) represents the 5th-95th percentile ranges for the Control group’s ERPs. This method allows for all six metrics to be plotted radially on the same scale, and to visualize multivariate change either within or between groups.

## 4 Results

**Figure 1:**
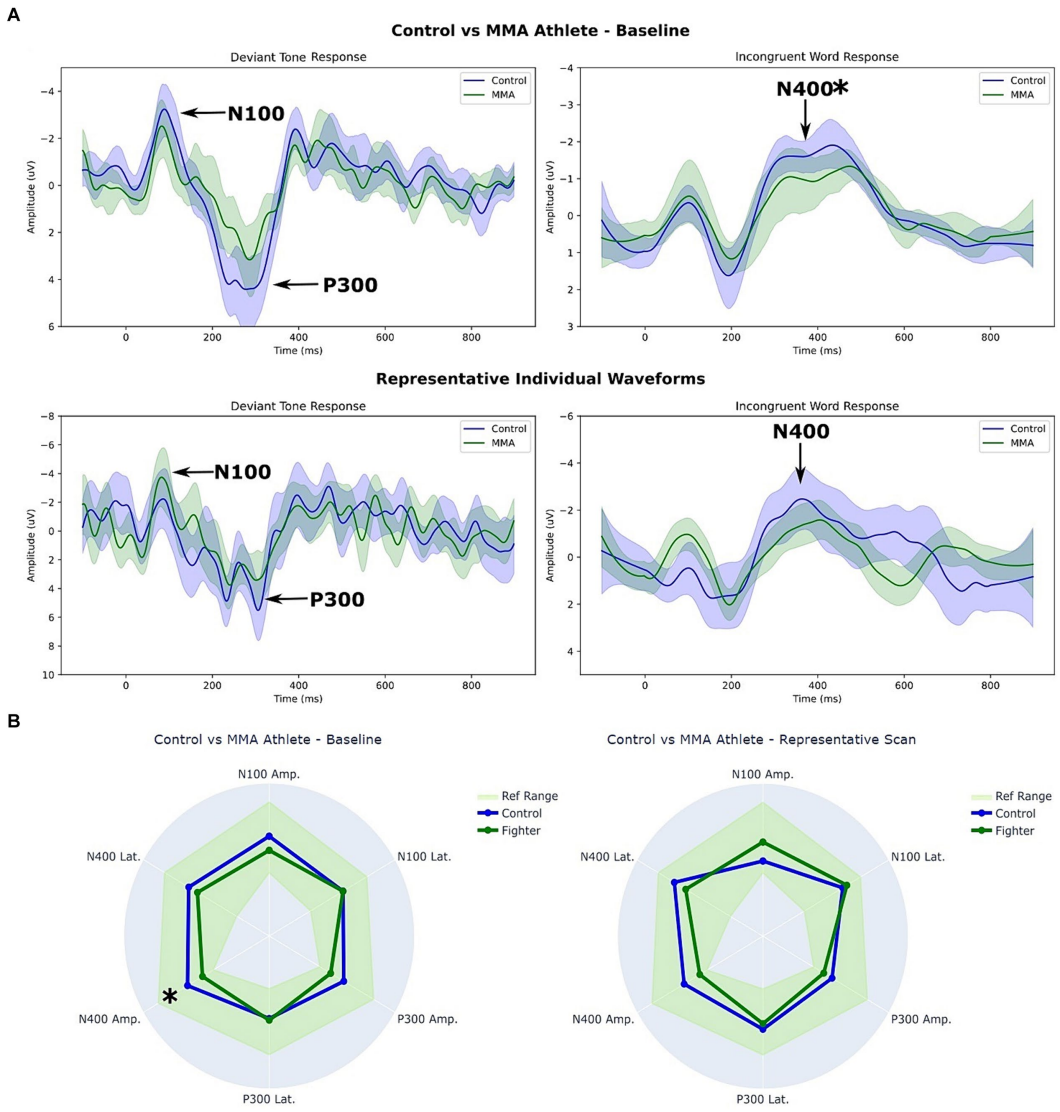
**(A)** Waveforms and **(B)** Radar plot comparison between MMA athletes and controls at baseline. * *p* < 0.05 (corrected). The shaded regions in **(A)** represent the 5th – 95th % confidence intervals for each waveform. The ref. range in **(B)** represents the 5th – 95th percentile ranges for the control groups ERPs.

Figure 1A depicts waveform results from the MMA athlete and Control groups. The first row, titled *Fighters vs Control – Baseline,* contrasts the average group responses to the Deviant tones and the Incongruent word pairs, respectively. The shaded regions represent the 5th – 95th % confidence intervals for each waveform. The second row, titled *Representative Individual Waveforms,* contrasts the response of a single participant (one Control vs. one MMA athlete) to the Deviant tones and Incongruent word pairs.

Figure 1B depicts two radar plots comparing peak amplitudes and latencies at the group level (left), and at the individual level (right). The same two participants’ data were used for the representative individual scan in Figures 1A,B.

A Supplementary Figure S1 is attached displaying the group-level average waveforms for each stimulus. While not significant, group differences were also detectable in the congruent condition, with a pronounced lack of separation in the MMA group relative to the Control group.

## 5 Discussion

The study investigated subconcussive changes in brain vital signs in MMA athletes compared to matched controls at baseline. The findings supported the hypothesis that MMA athletes would show significant brain vital sign differences relative to controls, presumably due to routine exposure to repetitive head impacts while participating in their sport. While the brain vital sign task is passive and did not require a response, the N100 is generally sensitive to discriminating louder deviant tones, the P300 is generally sensitive to attending to the unexpected occurrence of the louder deviant tones, and the N400 is generally sensitive to semantically discriminating a mismatching word pair (9).

Examination of the waveform differences between MMA athletes and controls showed a common reduction in N400 response amplitudes. The current findings contribute to the growing body of literature that supports the link between repetitive head impacts and subconcussive impairment. Given our recent ice hockey study (18), which demonstrated no detectable subconcussive impairments with specific daily dietary supplementation, future work should investigate potentially effective and accessible intervention options. Furthermore, frequency domain and resting state analyses were not conducted. However, future studies should explore possible EEG frequency and resting state effects.

The current study represents another replication in support of the relationship between head impact exposure and brain vital signs (12–14, 18). Specifically, subconcussive changes in the cognitive N400 response have repeatedly been observed across all studies to-date. The replications have been across different contact sports (i.e., ice hockey, football, and mixed martial arts) as well as different age ranges (i.e., approximately ages 12–30 years old) in male athletes. It is important to note that changes to the N400 (and often N100) are likely not the only changes, but rather the most common and consistent ones. Also, that the particular N400 changes have varied in terms of latency delays, amplitude reductions, or both across the noted studies. The two factors are interdependent and interactive, with relative peak timing delays and response reductions representing a relative impairment in cognitive processing, specifically semantic processing (19–21). While there are a number of different incongruent and/or congruent waveform changes that are associated with impaired semantic processing, and that the reduction in the incongruent N400 amplitudes represented a common possible changes frequently measured directly in the brain vital sign framework. On-going studies have further confirmed the N400 effects compared to non-contact control comparisons, across different sports, and between females and males (22).

## 6 Limitations

Our available subject pool of MMA athletes was limited, leading to a relatively low sample size. Moreover, we had to exclude some acquired EEG scans due to data quality, precluding us from conducting a planned longitudinal analysis. While subjects in the control group were not participating in contact sports during the study, other lifetime exposures were not accounted for that may have impacted our findings. Furthermore, we cannot differentiate if the measured baseline differences reflect an association with MMA participation in general, or the acute effects of recent MMA activity. Future studies with larger sample sizes will make it possible to evaluate specific factors related to MMA participation, such as fight history, outcomes and training methods that may have influenced our results. Moreover, future studies would benefit from incorporating fight performance metrics or using impact sensors to derive specific head impact measures of exposure. Likewise, a more robust longitudinal design would allow for direct assessment of brain vital sign changes over time in relation to MMA exposure measures.

## 7 Conclusion

MMA athletes demonstrate significant brain vital sign differences in baseline scans compared to controls. Specifically, significant brain vital sign differences were detected as reduced amplitudes for the N400, with additional reductions observed in the N100 and P300. The findings indicate that exposure to repetitive head impacts in MMA may be associated with subconcussive changes in brain function.

## Data Availability

The raw data supporting the conclusions of this article will be made available by the authors, without undue reservation.
