# Supplementary figures and images for: Mixed martial arts athletes demonstrate different brain vital sign profiles compared to matched controls at baseline
Source: Front Neurol. 2024 Sep 12;15:1438368. doi: 10.3389/fneur.2024.1438368 (PMC11448351; doi:10.3389/fneur.2024.1438368)

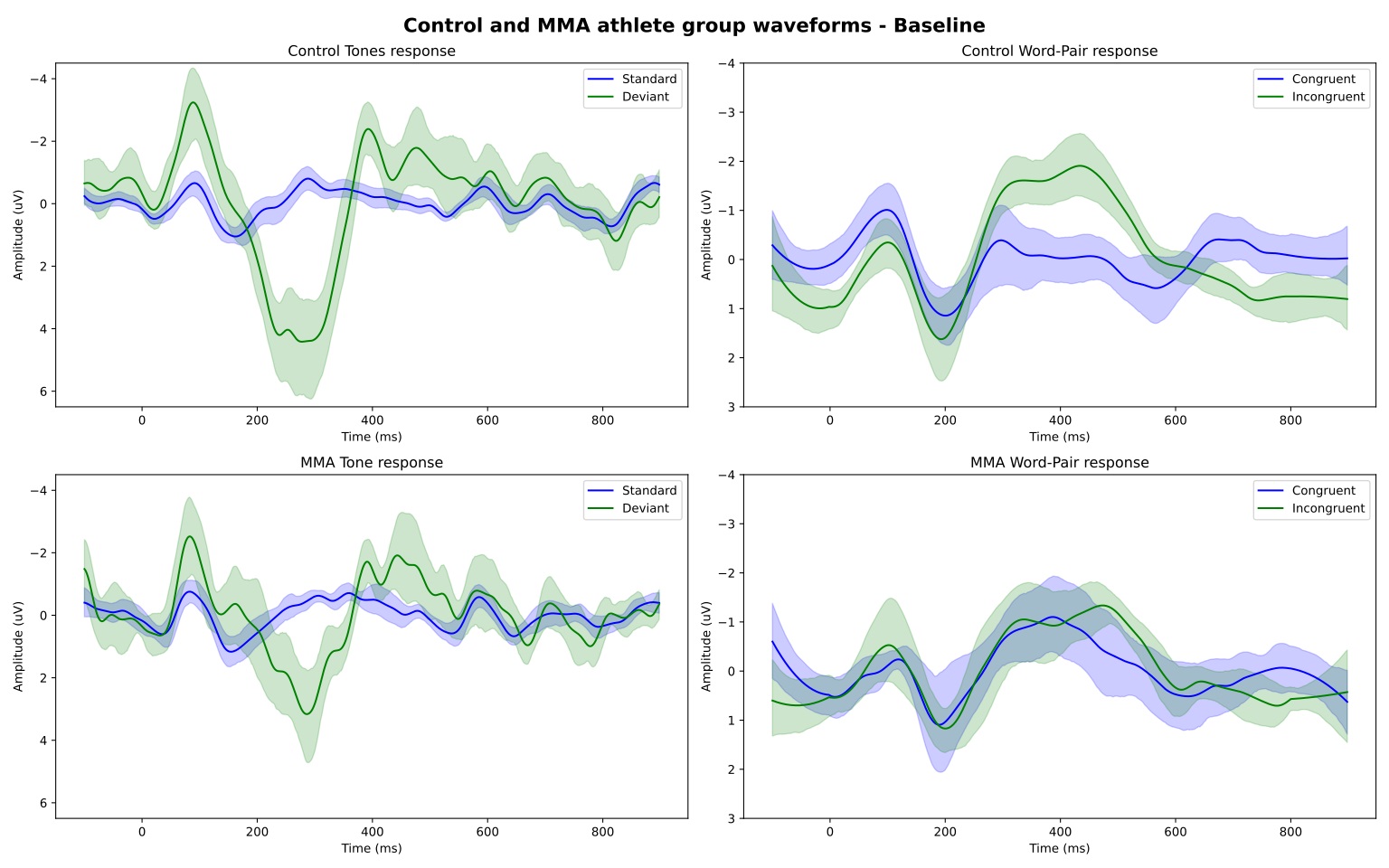

Supplement: Supplementary file 1 [file Image_1.JPEG]
